# Risk factor analysis and construction of prediction models of gallbladder carcinoma in patients with gallstones

**DOI:** 10.3389/fonc.2023.1037194

**Published:** 2023-02-27

**Authors:** Zhencheng Zhu, Kunlun Luo, Bo Zhang, Gang Wang, Ke Guo, Pin Huang, Qiuhua Liu

**Affiliations:** ^1^ Department of Hepatobiliary Surgery, Zhangjiagang City First People’s Hospital, Suzhou, China; ^2^ Department of Hepatobiliary Surgery, The 904th Hospital of Joint Logistic Support Force of PLA, Wuxi, China

**Keywords:** gallbladder neoplasms, risk factors, clinical prediction model, nomogram, logistic (logit) regression

## Abstract

**Background:**

Gallbladder carcinoma (GBC) is a biliary tract tumor with a high mortality rate. The objectives of this study were to explore the risk factors of GBC in patients with gallstones and to establish effective screening indicators.

**Methods:**

A total of 588 patients from medical centers in two different regions of China were included in this study and defined as the internal test samples and the external validation samples, respectively. We retrospectively reviewed the differences in clinicopathologic data of the internal test samples to find the independent risk factors that affect the occurrence of GBC. Then, we constructed three different combined predictive factors (CPFs) through the weighting method, integral system, and nomogram, respectively, and named them CPF-A, CPF-B, and CPF-C sequentially. Furthermore, we evaluated these indicators through calibration and DCA curves. The ROC curve was used to analyze their diagnostic efficiency. Finally, their diagnostic capabilities were validated in the external validation samples.

**Results:**

In the internal test samples, the results showed that five factors, namely, age (RR = 3.077, 95% CI: 1.731-5.496), size of gallstones (RR = 13.732, 95% CI: 5.937-31.762), course of gallstones (RR = 2.438, 95% CI: 1.350-4.403), CEA (RR = 9.464, 95% CI: 3.394-26.392), and CA199 (RR = 9.605, 95% CI: 4.512-20.446), were independent risk factors for GBC in patients with gallstones. Then, we established three predictive indicators: CPF-A, CPF-B, and CPF-C. These models were further validated using bootstrapping with 1,000 repetitions. Calibration and decision curve analysis showed that the three models fit well. Meanwhile, multivariate analysis showed that CPF-B and CPF-C were independent risk factors for GBC in patients with gallstones. In addition, the validation results of the external validation samples are essentially consistent with the internal test samples.

**Conclusion:**

Age (≤58.5 *vs*. >58.5 years), size of gallstones (≤1.95 *vs*. >1.95cm), course of gallstones (≤10 *vs*. >10 years), CEA (≤5 *vs*. >5 ng/ml), and CA199 (≤37 *vs*. >37 U/ml) are independent risk factors for GBC in patients with gallstones. When positive indicators were ≥2 among the five independent risk factors or the score of the nomogram was >82.64, the risk of GBC was high in gallstone patients.

## Introduction

Gallbladder carcinoma (GBC) is a highly malignant tumor of the biliary tract, accounting for 80%-95% of the global biliary tract tumors (1). The prognosis of GBC is poor, and the 5-year survival rate does not exceed 10% (2). Early diagnosis and early treatment are ideal treatment options. But patients with GBC only have non-specific biliary system symptoms such as right upper abdominal pain, nausea, jaundice, and fever, which are difficult to distinguish from the benign gallbladder diseases *via* symptoms. Only one-third of GBCs can be diagnosed before surgery, and the prognosis of patients who lose the chance of surgery is extremely poor (2). The effectiveness of traditional imaging examinations, such as abdominal ultrasound, enhanced computed tomography (CT), and enhanced magnetic resonance (MR), is not ideal for the diagnosis of early-stage GBC. Improved diagnostic strategies, such as endoscopic ultrasonography (EUS), DWI combined with T2WI, circulating tumor cell (CTC) examination, and PET-CT, have an increased diagnosis rate of GBC which is more than 80%-90%, but most of these tests are expensive and difficult to promote as a screening method in patients, which brings difficulties to the early diagnosis rate of GBC (3–7). Therefore, the improvement of early screening and systematic diagnosis strategies is particularly important for this deadly disease of GBC.

The mechanism of GBC is not yet fully understood. It has been agreed that the occurrence of GBC is influenced by many factors such as the environment, not just genetics (1, 8). Gallstones are the most important risk factor.

The inflammatory response induced by gallstones is an important mechanism of its inflammatory cancer transformation, but the specific relationship between gallstones and GBC is not clear yet (2). In addition, gender, age, obesity, diabetes, occupational exposure, smoking, and other factors have also been reported that may be related to the occurrence of GBC (3).

Therefore, this study was designed by analyzing and comparing the medical records of GBC patients and patients with gallstones diagnosed in a single center during the past 10 years, to identify independent risk factors for the occurrence of GBC in patients with gallstones and to establish three combined predictive factors through different methods. We further verified the diagnostic capabilities of these factors with external samples from another regional medical center, in order to find new effective indicators that are effective in the early screening of GBC.

## Methods

### Patients

**Figure 1:**
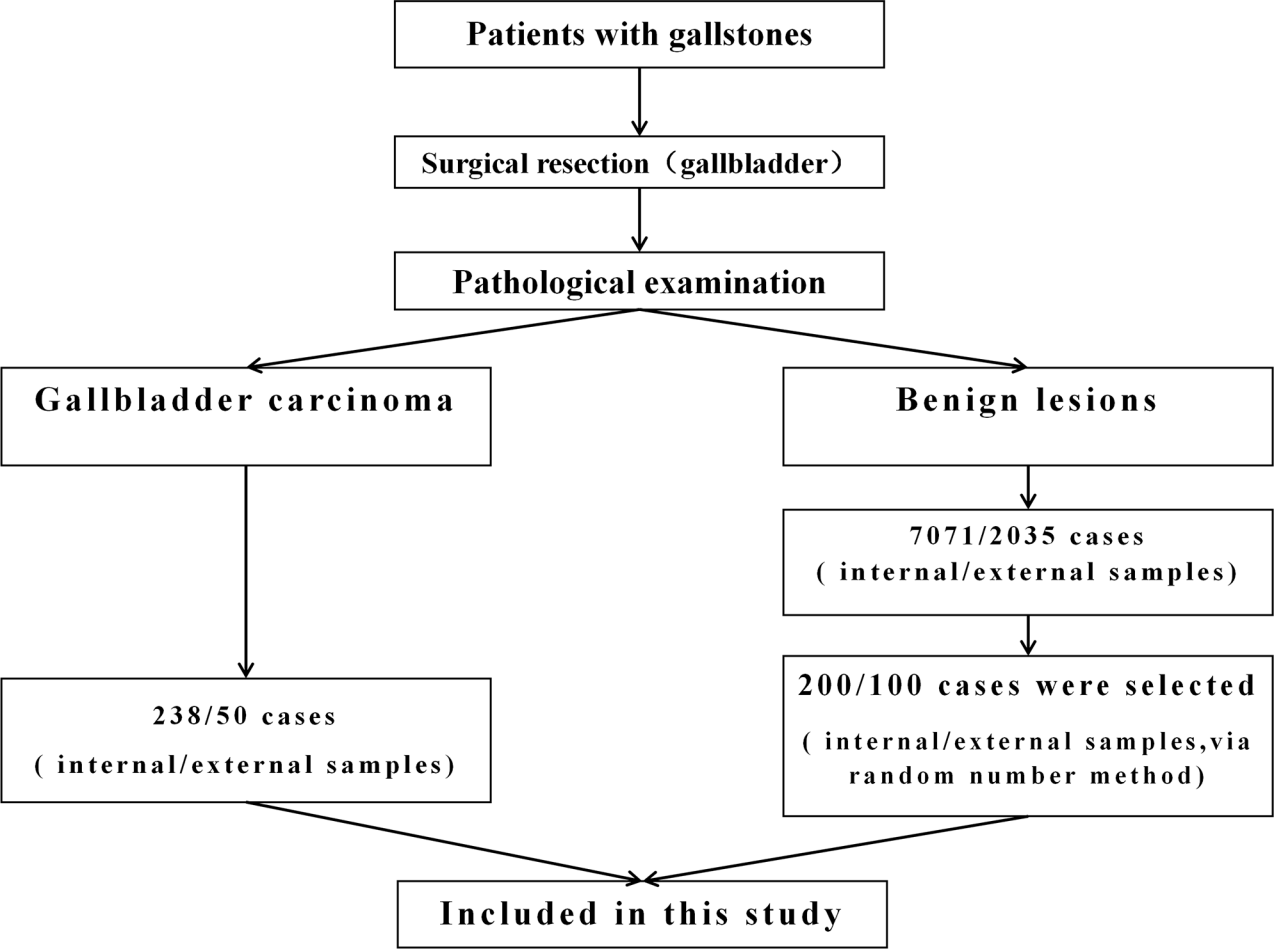
The flowchart to identify patients with gallbladder carcinoma (GBC) and gallstone (GS).

The patients included in this study were divided into internal test samples and external validation samples (**Figure 1**).

In the internal test samples, 238 patients (GBC group) who had a history of gallstones and were diagnosed with GBC through pathological examination at the 904th Hospital of Joint Logistic Support Force of PLA in Wuxi, China, from January 2010 to June 2020 were enrolled. The data at the time of diagnosis of GBC were collected. All GBC patients underwent at least cholecystectomy to clarify the data of gallbladder and gallstones. In addition, among the 7,071 patients with gallstones who underwent laparoscopic cholecystectomy during the same period, 200 patients (GS group) were randomly selected by the random number method, and their medical records were collected. The postoperative pathological examination showed that the gallbladder of these patients had benign lesions.

In the external validation samples, 50 patients (GBC group) who had a history of gallstones and were diagnosed as GBC through pathological examination at Zhangjiagang City First People’s Hospital in Suzhou, China, from January 2019 to August 2022 were enrolled. The data at the time of diagnosis of GBC were collected. In addition, among the patients with gallstones who underwent laparoscopic cholecystectomy during the same period, 100 patients (GS group) were randomly selected by the random number method. The postoperative pathological examination showed that the gallbladder of these patients had benign lesions.

The exclusion criteria were as follows (9, 10): 1) patients with incomplete medical records or GBC patients not sure of having a history of gallstones; 2) patients who have not undergone cholecystectomy or whose postoperative pathology cannot clearly determine the condition of gallstones and gallbladder; 3) patients with other tumor diseases; 4) patients previously treated with corticosteroids or with hematological or oncological diseases; 5) unclear pathological diagnosis, like perihilar cholangiocarcinoma extended to the gallbladder and GBC extended to the hilum; 6) patients with gallbladder polyps; 7) patients with diseases clearly related to GBC which had surgical indications, such as porcelain gallbladder, Mirizzi syndrome, and pancreaticobiliary anatomy; and 8) patients with bile duct stones.

### Research indicators

**Figure 2:**
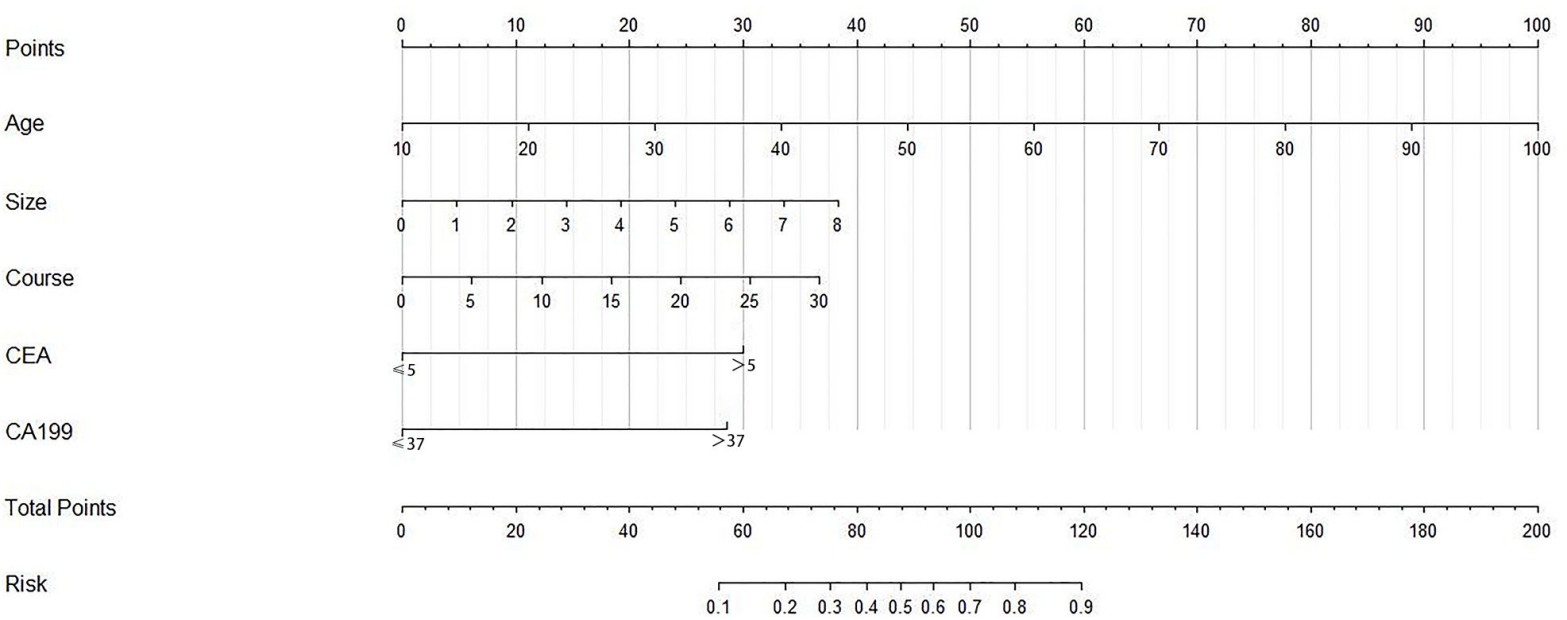
The nomogram model for predicting the risk of GBC in patients with gallstones. CEA, carcinoembryonic antigen (ng/ml); CA199, carbohydrate antigen 199 (U/ml); Size, size of gallstones (cm); Course, course of gallstones (year).

1. General information: age, gender, BMI, blood type, comorbidities (hypertension, diabetes);
2. Environmental factors:
a. Smoking, drinking;
b. Occupational exposure (3), such as rubber, textile, petroleum, and shoe factories;
3. Features of gallbladder and gallstones:
a. Confirm the course of gallstones by telephone (years), which starts from the time when gallstones were first discovered and ends with the time of surgical removal of the gallbladder;
b. Confirm the size of gallstones (cm) through the pathology after the patient’s operation. The diameter of the stone = (long diameter + short diameter)/2; the spherical diameter of multiple stones or sediment-like stones was estimated according to their total volume;
4. Preoperative laboratory examination: Collect peripheral blood indicators of patients from the medical record system of two hospitals at the time of admission during surgery, including CRP, white blood cell, neutrophil count, lymphocyte count, albumin, CEA, and CA199;
5. Combined predictive factor (CPF): Based on the results of logistic multivariate analysis, three methods, namely, the weighting method, integral method, and nomogram, were used to construct the indicators. a) According to logistic analysis regression coefficient (*B*), with minimum *B*_min_ as the benchmark, the value of each influencing factor (*X*) = *B_x_*/*B*_min_. The sum of the independent risk factors’ values was CPF-A. b) According to the independent risk factors shown in the multivariate analysis results, CPF-B = the number of positive independent risk factors. c) Based on the results of logistic multivariate analysis, the nomogram was constructed by the R software and assigned to each patient (**Figure 2**). The value of each patient was recorded as CPF-C. Meanwhile, based on the cutoff values of the ROC curves of three different CPF models, the patients were divided into positive and negative groups. The proportion of GS patients in the positive group was the misdiagnosis rate of this indicator. The proportion of GBC patients in the negative group was the missed diagnosis rate of this indicator.

### Statistical analysis

The data were analyzed using SPSS 23.0, MedCalc v19.0.7, and R 4.2.1. The Kolmogorov–Smirnov test was used to test the normality of continuous variables. Variables conforming to normal distribution were analyzed using *t*-test. Non-normal data were expressed by the Mann–Whitney *U* test. Categorical variables were analyzed by the chi-square test or Fisher’s exact test. Univariate analysis between different groups was performed. The ROC curve was established to determine the optimal critical value of different factors. Combining the results of univariate analysis, factors that may have an impact on the occurrence of GBC were incorporated into the logistic model (LR: forward) for multivariate analysis to find independent risk factors. Furthermore, the combined predictive factors were established through the weighting method, integration method, and nomogram. Then, the model was verified through the calibration curve and decision curve analysis (DCA) (bootstrap = 1,000). The critical value and diagnostic efficiency were analyzed by the ROC curve, and the AUC value of different indicators was compared by the MedCalc software. Finally, the prediction model was reintroduced into the multivariate analysis. A *P*-value <0.05 was considered statistically significant.

## Results

### Basic characteristics of the 438 gallstone patients that constituted the internal test samples

Among the 438 patients, 167 were men (38.1%) and 271 were women (61.9%), with a median age of 63 years (range 17-96 years). Among the 238 GBC patients, 221 patients (92.9%) had symptoms at the time of consultation, of which 200 patients presented with different degrees of right upper abdominal pain and 21 patients presented with symptoms such as jaundice, fatigue, or anorexia. Seventeen GBC patients (7.1%) had no symptoms, and they were diagnosed accidentally during physical examination or with other diseases. Among the 200 patients with gallstones, 198 cases had different degrees of abdominal pain at the time of consultation. Two patients were asymptomatic at the time of consultation, but surgical resection was performed because the diameter of the gallstone was greater than 2 cm. The postoperative pathology of these patients with gallstones all showed benign lesions with various degrees of inflammation. All patients underwent abdominal ultrasound examination before surgery. Among GBC patients, 148 patients were found to have a gallbladder mass by abdominal ultrasound, accounting for 62.2% of the patients with GBC. There were 223 cases who received enhanced CT examination, and 166 cases were positive (74.4%). On the other hand, 213 cases received enhanced MR examination, and 145 cases were positive (68.1%).

### Comparison of the clinicopathologic characteristics between GBC and GS in the internal test samples

Comparing the data of 238 GBC patients (GBC) and 200 patients with gallstones (GS), the results showed that there were no significant differences in gender, blood type, comorbidities (diabetes and hypertension), drinking, occupational exposure, gallbladder atrophy, and TBIL between the two groups (*P* > 0.05, see **Table 1**), but there were some differences in other aspects. The patients in the GBC group were significantly older than those in the GS group (68.0 ± 11.6 *vs*. 52.6 ± 13.3 years, *P* < 0.001). In addition, there was a difference in BMI between the GBC group and the GS group (22.2 ± 3.8 *vs*. 23.3 ± 3.4, *P* = 0.004). The number of smokers in the GBC group was significantly higher than that in the GS group (4.2% *vs*. 0.5%, *P* = 0.014). In terms of features related to gallstones, the size of gallstones in the GBC group was significantly larger (median 3.0 *vs*. 2.1 cm, *P* < 0.001), and the GBC group had a longer course of gallstones than the GS group (>10 *vs*. ≤10 years, *P* < 0.001). The laboratory examination results showed that inflammation-related indicators, such as leukocyte (median 6.8 *vs*. 5.7 × 10^9^/L, *P* < 0.001), neutrophils (median 4.6 *vs*. 3.4 × 10^9^/L, *P* < 0.001), lymphocytes (median 1.3 *vs*. 1.8 × 10^9^/L, *P* < 0.001), and CRP (median 17.3 *vs*. 1.7 mg/L, *P* < 0.001), were significantly different in the GBC group compared with those in the GS group. Meanwhile, the tumor indicators CEA (median 4.0 *vs*. 1.8 μg/L, *P* < 0.001) and CA199 (median 63.7 *vs*. 10.9 U/ml, *P* < 0.001) had higher values in the GBC group than in the GS group. In addition, there were also significant differences between the two groups in terms of ALB, ALT, AST, γ-GT, ChE, ALP, etc. (*P* < 0.05, **Table 1**).

**Table 1:** Comparison of general data between GBC and GS in the internal test samples. .

Variable	GBC (*n* = 238, 54.3%)	GC (*n* = 200, 45.7%)	*χ* ^2^/*t* (U)	*P*-value
Gender			0.294	0.588
Male	88 (37.0)	79 (39.6%)		
Female	150 (63.0)	121 (60.5%)		
Blood type			1.640	0.650
A	73 (30.7)	69 (34.5)		
B	70 (29.4)	59 (29.5)		
O	60 (25.2)	50 (25.0)		
AB	35 (14.7)	22 (11.0)		
Occupational exposure^a^	40 (16.8)	25 (12.5)	1.595	0.207
Smoking^b^	10 (4.2)	1 (0.5)	6.082	0.014
Drinking^c^	5 (2.1)	3 (1.5)	0.219	0.732
Hypertension	72 (30.3)	47 (23.5)	2.504	0.114
Diabetes	30 (12.6)	24 (12.0)	0.037	0.848
Course of gallstones (years)			38.828	<0.001
<1	26 (10.9)	53 (26.5)		
1~10	107 (45.0)	110 (55.0)		
>10	105 (44.1)	37 (18.5)		
Gallbladder atrophy	18 (7.6)	15 (7.5)	0.001	0.980
Age (years)	68.0 ± 11.6	52.6 ± 13.3	−12.813	<0.001
BMI	22.2 ± 3.8	23.3 ± 3.4	2.857	0.004
Size of gallstones (cm)	3.0 (2.3-3.9)	2.1 (1.2-3.3)	32,252.500	<0.001
CRP (mg/L)	17.3 (3.9-50.4)	1.7 (1.0-4.0)	39,169.500	<0.001
Leukocytes (×10^9^/L)	6.8 (5.4-9.1)	5.7 (4.6-6.8)	31,889.500	<0.001
Neutrophils (×10^9^/L)	4.6 (3.4-6.5)	3.4 (2.6-4.2)	34,292.500	<0.001
Lymphocytes (×10^9^/L)	1.3 (0.9-1.8)	1.8 (1.4-2.1)	15,094.500	<0.001
ALB (g/L)	36.4 (31.7-41.0)	42.4 (39.3-44.6)	11,399.500	<0.001
CEA (ng/ml)	4.0 (1.8-18.3)	1.8 (1.2-2.7)	35,144.500	<0.001
CA199 (U/ml)	63.7 (13.2-549.4)	10.9 (6.0-18.7)	37,674.000	<0.001
ALT (U/L)	27.5 (15.0-74.5)	21.0 (14.0-33.0)	27,663.000	0.003
AST (U/L)	27.5 (18.0-61.5)	19.0 (16.0-26.0)	31,748.000	<0.001
γ-GT (U/L)	88.5 (28.0-302.8)	29.5 (19.0-53.8)	33,458.000	<0.001
TBIL (μmol/L)	16.2 (10.7-36.8)	15.7 (12.0-20.2)	25,736.000	0.142
ChE (U/L)	135.0 (82.5-4371.0)	8,739.0 (7,613.8-10,344.3)	3,601.000	<0.001
ALP (U/L)	111.0 (74.0-246.5)	70.0 (57.3-94.0)	35,495.000	<0.001

Categorical data were presented as numbers (percentage); continuous data in normal distribution were presented as mean ± SD; continuous data in skewed distribution were presented as median (interquartile distance).

GBC, gallbladder carcinoma patients with preexisting gallstones; GS, patients with gallstones; TBIL, total bilirubin; ALB, albumin; CRP, C-reactive protein; CEA, carcinoembryonic antigen; CA199, carbohydrate antigen 199; ALT, alanine aminotransferase; AST, aspartate aminotransferase; γ-GT, γ-glutamyl transpeptidase; ALP, alkaline phosphatase.

a Industrial/occupational exposure related to GBC, including rubber, textiles, petroleum, and shoe factories (3)

b Smoking: ≥10 cigarettes/day.

c Drinking: ≥250 g/day.

### Analysis of risk factors of the occurrence of GBC in patients with gallstones in the internal test samples

After establishing the ROC curve, the results showed that the cutoff value of age was 58.5 years (95% CI: 0.761~0.841), and the cutoff value of the size of gallstones was 1.95 cm (95% CI: 0.641~0.745). Furthermore, although the univariate analysis showed that the inflammation-related indicators leukocytes, neutrophils, lymphocytes, and CRP and the liver injury-related indicators albumin, ALT, AST, γ-GT, ChE, and ALP were significantly different between GBC and GS patients, these indicators were not included in the multivariate analysis because the significance of these indicators in tumor diagnosis was not definite and their increase may be caused by tumor-necrotizing inflammation caused by GBC itself or liver tissue damage around the gallbladder. Therefore, we finally included age, BMI, smoking, size of gallstones, course of gallstones, CEA, CA199, and other indicators into the logistic model for multivariate analysis. The results of the multivariate analysis showed that age (RR = 3.077, 95% CI: 1.731-5.496), size of gallstones (RR = 13.732, 95% CI: 5.937-31.762), course of gallstones (RR = 2.438, 95% CI: 1.350~4.403), CEA (RR = 9.464, 95% CI: 3.394-26.392), and CA199 (RR = 9.605, 95% CI: 4.512-20.446) were independent risk factors for GBC in patients with gallstones(*P* < 0.05, **Table 2**).

**Table 2:** Logistic multivariate analysis of the risk factors associated with GBC in patients with gallstones in the internal test samples.

Variable	*B*	Standard error	Wald	*df*	*P*-value	Relative risk	95% CI
Age (≤58.5 *vs*. >58.5 years)	1.124	0.293	14.667	1	<0.001	3.077	1.731~5.496
Size of gallstones (≤1.95 *vs*. >1.95 cm)	2.620	0.428	37.490	1	<0.001	13.732	5.937~31.762
Course of gallstones (≤10 *vs*. >10 years)	0.891	0.301	8.740	1	0.003	2.438	1.350~4.403
CEA (≤5 *vs*. >5 ng/ml)	2.248	0.523	18.450	1	<0.001	9.464	3.394~26.392
CA199 (≤37 vs. >37 U/ml)	2.262	0.385	34.446	1	<0.001	9.605	4.512~20.446

Factors included in the model include age, BMI, smoking, size of gallstones, course of gallstones, CEA, and CA199; all factors were included in the study as categorical variables (LR: forward).

### Establishment of the combined predictive factors for GBC through different models in the internal test samples

First, through the weighting method, according to the logistic analysis regression coefficient (*B*), with the minimum *B*_min_ (course of gallstones) = 0.891 as the benchmark, the value of each influencing factor (*X*) = *B_x_*/*B*_min_ (for the convenience of calculation, the multiples of 0.5 are taken). Thus, the model was obtained: CPF-A = (age * 1 + size of gallstones * 3 + course of gallstones * 1 + CEA * 2.5 + CA199*2.5), and it was assigned to each patient in the internal test samples.

**Figure 3:**
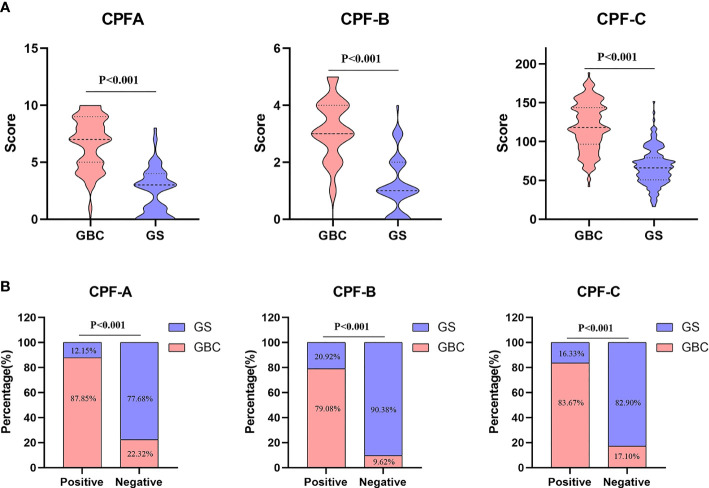
Data of combined predictive factor (CPF)-A, CPF-B, and CPF-C. **(A)** Comparison of CPF-A, CPF-B, and CPF-C scores between GBC and GS in the internal test samples. **(B)** Diagnosis rate of CPF-A, CPF-B, and CPF-C in the internal test samples.

**Figure 4:**
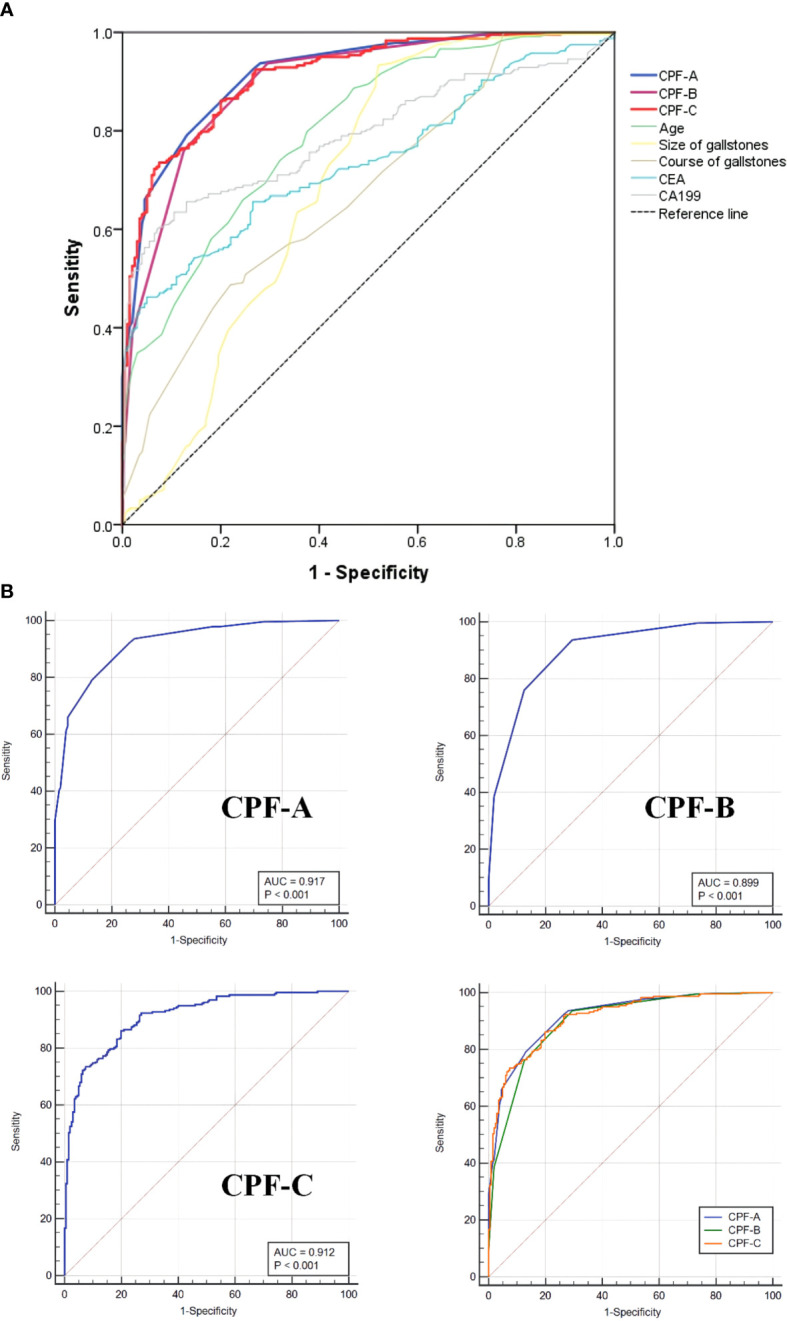
The ROC curves of different factors in the internal test samples. **(A)** The ROC curves of CPF-A, CPF-B, CPF-C and other risk factors associated with GBC. **(B)** The ROC curves for assessing the discrimination performance of CPF-A, CPF-B and CPF-C.

The CPF-A scores of the GBC group and the GS group were compared. The results showed that the score of the GBC group was significantly higher than that of the GS group (*P* < 0.001, **Figure 3A**). The ROC curve was further established, and the cutoff value of CPF-A was 4.25. The AUC value at this time was 0.917 (95% CI: 0.892-0.943), the sensitivity was 0.790, and the specificity was 0.870 (**Table 3**, **Figure 4B**). Based on the cutoff value, patients in the internal test samples were divided into the CPF-A-positive group (positive, CPF-A > 4) and the CPF-A-negative group (negative, CPF-A ≤ 4). At this time, there were 214 patients in the CPF-A-positive group, consisting of 188 GBC patients (87.85%) and 26 GS patients (12.15%), and 224 patients in the CPF-A-negative group, consisting of 50 GBC patients (22.32%) and 174 GS patients (77.68%). Therefore, in the internal test samples, the misdiagnosis rate of CPF-A was 12.15%, and the missed diagnosis rate was 22.32% (**Figure 3B**).

**Table 3:** Predictive values of predictive factors associated with GBC in the internal test samples.

Variable	AUC	Cutoff value	Sensitivity	Specificity	Standard error	95% CI	*P*-value
CPF-A	0.917	4.25	0.790	0.870	0.013	0.892~0.943	<0.001
CPF-B	0.899	1.50	0.937	0.705	0.015	0.870~0.928	<0.001
CPF-C	0.912	82.64	0.861	0.800	0.013	0.886~0.938	<0.001
Age	0.801	58.5	0.798	0.625	0.020	0.761~0.841	<0.001
Size of gallstones	0.693	1.95	0.933	0.480	0.027	0.641~0.745	<0.001
Course of gallstones	0.628	0.50	0.441	0.815	0.027	0.576~0.680	<0.001
CEA	0.738	4.5	0.462	0.950	0.023	0.692~0.784	<0.001
CA199	0.791	32.87	0.601	0.930	0.022	0.749~0.834	<0.001

Then, through the point system, among the independent risk factors of GBC, positive ones were scored 1 point, negative ones were scored 0 points, and the CPF-B was obtained. Comparing the CPF-B between the GBC group and the GS group, the results showed that there was a statistically significant difference between the two groups (*P* < 0.001, **Figure 3A**). The ROC curve was further established, and the cutoff value of CPF-B was 1.50. The AUC value at this time was 0.899 (95% CI: 0.870-0.928), the sensitivity was 0.937, and the specificity was 0.705 (**Table 3**, **Figure 4B**). Based on the cutoff value, patients that constituted the internal test samples were divided into the CPF-B-positive group (positive, CPF-B ≥ 2) and the CPF-B-negative group (negative, CPF-B < 2). At this time, there were 282 patients in the CPF-B-positive group, consisting of 233 GBC patients (79.08%) and 59 GS patients (20.92%), and 156 patients in the CPF-B-negative group, consisting of 15 GBC patients (9.62%) and 141 GS patients (90.38%). Therefore, in the internal test samples, the misdiagnosis rate of CPF-B was 20.92%, and the missed diagnosis rate was 9.62% (**Figure 3B**).

Based on the results of the logistic regression analysis, we established the nomogram through the R software (**Figure 2**) and then assigned it to each patient according to the nomogram map, recorded as CPF-C. Comparing the CPF-C between the GBC group and the GS group, the results also showed that there was a statistically significant difference between the two groups (*P* < 0.001, **Figure 3A**). The ROC curve was further established, and the cutoff value of CPF-C was 82.64.

The AUC value at this time was 0.912 (95% CI: 0.886-0.938), the sensitivity was 0.861, and the specificity was 0.800 (**Table 3**, **Figure 4B**). Based on the cutoff value, patients that constituted the internal test samples were divided into the CPF-C-positive group (positive, CPF-C > 82.64) and the CPF-C-negative group (negative, CPF-C ≤ 82.64). At this time, there were 245 patients in the CPF-C-positive group, consisting of 205 GBC patients (83.67%) and 40 GS patients (16.33%), and 193 patients in the CPF-C-negative group, consisting of 33 GBC patients (17.1%) and 160 GS patients (82.9%). Therefore, in the internal test samples, the misdiagnosis rate of CPF-B was 16.33%, and the missed diagnosis rate was 17.1% (**Figure 3B**).

### The evaluation of CPF-A, CPF-B, and CPF-C in the internal test samples

**Figure 5:**
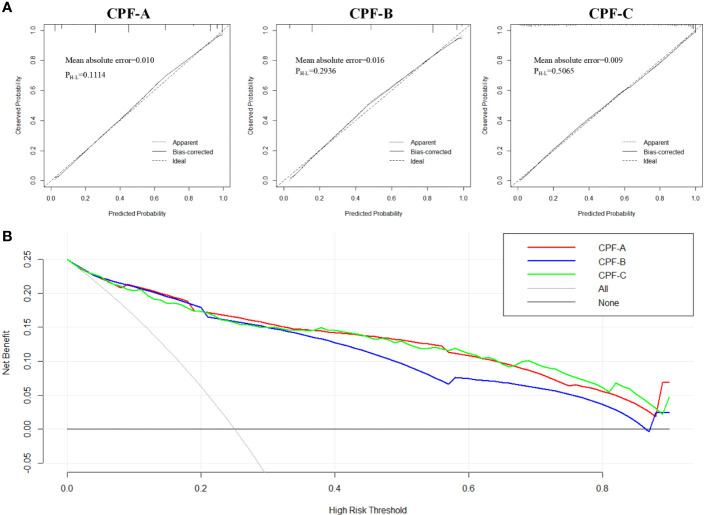
The evaluation of CPF-A, CPF-B, and CPF-C in the internal test samples. **(A)** Calibration curves of high-risk patients for GBC predicted by CPF-A, CPF-B, and CPF-C. **(B)** Decision curves of CPF-A, CPF-B, and CPF-C.

Next, we evaluated the diagnostic values of the three models in the internal test samples *via* the R software. The C-index values of CPF-A, CPF-B, and CPF-C were 0.917, 0.899, and 0.912, respectively, which were the same as their AUC values. The corrected C-index values of CPF-A, CPF-B, and CPF-C were 0.916, 0.900, and 0.909, which proved that the prediction accuracy of the three indicators was high. Furthermore, by establishing the calibration curve (bootstrap = 1,000, *n* = 438), the results showed that the models of the three predictors were well fitted. At this time, the mean absolute errors of CPF-A, CPF-B, and CPF-C were 0.010, 0.016, and 0.009, respectively. Meanwhile, through the Hosmer–Lemeshow analysis, the *P*-values of CPF-A, CPF-B, and CPF-C were 0.1114, 0.2936, and 0.5065, respectively (*P* > 0.05, **Figure 5A**). These results prove that the fitted value of the three models were all in good agreement with the observed value, and the fitting degree is excellent.

Then, we compared the diagnostic benefits of the three models through DCA. The results showed that within the threshold of approximately 0.3-0.8, the net benefit of CPF-A and CPF-C was higher than that of CPF-B (**Figure 5B**). Furthermore, the clinical impact curve showed that when the thresholds were approximately 0.4 or higher, the CPF-A and CPF-C models predicted a high degree of agreement between the number of positives and the true number of GBC. For the prediction model CPF-B, when the threshold value was above 0.6, its prediction accuracy was higher (**Supplementary Material 1**).

Moreover, we included the three prediction models in the ROC curve to compare their AUC values. The results showed that the AUC value of CPF-A was significantly higher than that of CPF-B (*P* < 0.001). But there was no significant difference in AUC value between CPF-B and CPF-C or between CPF-B and CPF-A (*P* > 0.05). The AUC values of the ROC curves of the three indicators and five independent risk factors (age, size of gallstones, course of gallstones, CEA, CA199) were compared. The results showed that the AUC value of the three combined predictors was significantly higher than that of the other five independent risk factors (*P* < 0.05, **Figure 4A**, **Table 3**).

Finally, we included the three combined predictors and the seven risk factors, namely, age, BMI, smoking, size of gallstones, course of gallstones, CEA, and CA199, into the logistic model again for multivariate analysis in the internal test samples. The results showed that when CPF-A (≤4 *vs*. >4) was included, its independent risk factors were age, size of gallstones, course of gallstones, CEA, and CA199 (*P* < 0.05, **Table 4**), while CPF-A was not an independent factor of GBC (*P* > 0.05). However, when CPF-B (<2 *vs*. ≥2) or CPF-C (≤82.64 *vs*. >82.64) was included, they were still the independent risk factors for GBC (*P* < 0.05, **Table 4**).

**Table 4:** Multivariate analysis after incorporating CPF-A, CPF-B, and CPF-C in the internal test samples.

Variable	*B*	Standard error	Wald	*df*	*P*-value	Relative risk	95% CI
Incorporating CPF-A (≤4 *vs*. >4)							
Age (≤58.5 *vs*. >58.5 years)	1.124	0.293	14.667	1	<0.001	3.077	1.731~5.496
Size of gallstones (≤1.95 *vs*. >1.95 cm)	2.620	0.428	37.490	1	<0.001	13.732	5.937~31.762
Course of gallstones (≤10 *vs*. >10 years)	0.891	0.301	8.740	1	0.003	2.438	1.350~4.403
CEA (≤5 *vs*. >5 ng/ml)	2.248	0.523	18.450	1	<0.001	9.464	3.394~26.392
CA199 (≤37 *vs*. >37 U/ml)	2.262	0.385	34.446	1	<0.001	9.605	4.512~20.446
Incorporating CPF-B (<2 *vs*. ≥2)							
Size of gallstones (≤1.95 *vs*. >1.95 cm)	2.077	0.462	20.217	1	<0.001	7.984	3.228~19.747
CEA (≤5 *vs*. >5 ng/ml)	1.923	0.514	13.981	1	<0.001	6.840	2.497~18.742
CA199 (≤37 *vs*. >37 U/ml)	1.770	0.389	20.663	1	<0.001	5.869	2.736~12.588
CPF-B (<2 *vs*. ≥2)	1.864	0.353	27.842	1	<0.001	6.449	3.227~12.888
Incorporating CPF-C (≤82.64 *vs*. >82.64)							
Age (≤58.5 *vs*. >58.5 years)	0.782	0.340	5.272	1	0.022	2.185	1.121~4.257
Size of gallstones (≤1.95 *vs*. >1.95 cm)	2.477	0.434	32.564	1	<0.001	11.902	5.084~27.865
CEA (≤5 *vs*. >5 ng/ml)	1.867	0.527	12.560	1	<0.001	6.471	2.304~18.175
CA199 (≤37 *vs*. >37 U/ml)	1.716	0.416	16.985	1	<0.001	5.564	2.460~12.584
CPF-C (≤82.64 *vs*. >82.64)	1.071	0.379	7.992	1	0.005	2.918	1.389~6.129

In addition to CPF-A, CPF-B, and CPF-C, factors included in the model include age, BMI, smoking, size of gallstones, course of gallstones, CEA, and CA199; all factors were included in the study as categorical variables (LR: forward).

### Analysis of risk factors for the occurrence of GBC in patients with gallstones in the external validation samples

The CPF-A, CPF-B, and CPF-C obtained in the internal test samples were incorporated into the external validation samples, and the clinical data of these patients were collected. Comparing the 50 GBC patients with the 100 GS patients in the external validation samples, there were significant differences in clinicopathological factors such as age, size of gallstones, course of gallstones, BMI, CEA, and CA199 (*P* < 0.05, **Table 5**). Additionally, based on the criteria of the three predictor values obtained in the internal test samples, the results showed that CPF-A (median 4.3 *vs*. 3.0, *P* < 0.001), CPF-B (median 2.0 *vs*. 1.0, *P* < 0.001), and CPF-C (median 97.0 *vs*. 61.6, *P* < 0.001) of the GBC group were significantly higher than those of the GS group in the external validation samples (*P* < 0.05, **Table 5**).

**Table 5:** Comparison of general data between GBC and GS in the external validation samples.

Variable	GBC (*n* = 50, 33.33%)	GS (*n* = 10, 66.67%)	*χ* ^2^/*t* (U)	*P*-value
Gender			0.521	0.470
Male	16 (32.0)	38 (38.0)		
Female	34 (68.0)	62 (62.0)		
Blood type			0.485	0.922
A	17 (34.0)	37 (37.0)		
B	12 (24.0)	22 (22.0)		
O	15 (30.0)	32 (32.0)		
AB	6 (12.0)	9 (9)		
Occupational exposure^a^	3 (6.0)	7 (7.0)	0.054	0.817
Smoking^b^	5 (10.0)	11 (11.0)	0.035	0.851
Hypertension	15 (30.0)	36 (36.0)	0.535	0.465
Diabetes	5 (10.0)	12 (12.0)	0.133	0.716
Course of gallstones (years)			10.313	0.001
≤10	41 (82.0)	98 (98.0)		
>10	9 (18.0)	2 (2.0)		
Age (years)	65.9 ± 12.4	53.0 ± 15.5	−5.133	<0.001
BMI	21.9 ± 2.4	19.7 ± 2.1	−5.635	<0.001
Size of gallstones (cm)	2.5 (2.0-3.0)	1.9 (1.0-3.0)	3,465.000	<0.001
CEA (ng/ml)	2.3 (1.5-3.4)	1.6 (1.1-2.3)	3,421.500	<0.001
CA199 (U/ml)	16.0 (7.6-79.2)	10.4 (4.7-15.6)	3,522.000	<0.001
CPF-A	4.3 (4.0-6.5)	3.0 (0.0-3.0)	4,379.500	<0.001
CPF-B	2.0 (2.0-3.0)	1.0 (0.0-1.0)	4,362.500	<0.001
CPF-C	97.0 (80.7-122.2)	61.6 (45.3-76.2)	4,272.000	<0.001

Categorical data were presented as numbers (percentage); continuous data in normal distribution were presented as mean ± SD; continuous data in skewed distribution were presented as median (interquartile distance).

GBC, gallbladder carcinoma patients with preexisting gallstones; GS, patients with gallstones; CRP, C-reactive protein; CEA, carcinoembryonic antigen; CA199, carbohydrate antigen 199.

a Industrial/occupational exposure related to GBC, including rubber, textiles, petroleum, and shoe factories (3).

b Smoking: ≥10 cigarettes/day.

Next, we included age, size of gallstones, course of gallstones, BMI, CEA, CA199, and three CPFs into the logistic model for multivariate analysis. After excluding collinearity factors, the results showed that when CPF-A was included (≤4 *vs*. >4), its independent risk factors were age, size of gallstones, course of gallstones, and CA199 (*P* < 0.05, **Table 6**), while CPF-A was not an independent risk factor for GBC (*P* > 0.05). However, when CPF-B (<2 *vs*. ≥2) or CPF-C (≤82.64 *vs*. >82.64) was included, they were still the independent risk factors for GBC (*P* < 0.05, **Table 6**).

**Table 6:** Multivariate analysis of the risk factors associated with GBC in patients with gallstones in the external validation samples.

Variable	*B*	Standard error	Wald	*df*	*P*-value	Relative risk	95% CI
Incorporating CPF-A (≤4 *vs*. >4)							
Age (≤58.5 *vs*. >58.5 years)	1.561	0.479	10.617	1	0.001	4.765	1.863~12.187
Size of gallstones (≤1.95 *vs*. >1.95 cm)	2.321	0.644	12.975	1	<0.001	10.184	2.881~36.002
Course of gallstones (≤10 *vs*. >10 years)	2.673	1.013	6.957	1	0.008	14.486	1.987~105.594
CA199 (≤37 *vs*. >37 U/ml)	3.267	0.810	16.274	1	<0.001	26.238	5.365~128.327
Incorporating CPF-B (<2 *vs*. ≥2)							
Size of gallstones (≤1.95 *vs*. >1.95 cm)	1.651	0.702	5.537	1	0.019	5.214	1.318~20.634
Course of gallstones (≤10 *vs*. >10 years)	2.066	1.005	4.228	1	0.040	7.893	1.102~56.545
CA199 (≤37 *vs*. >37 U/ml)	2.698	0.808	11.142	1	0.001	14.844	3.045~72.353
CPF-B (<2 *vs*. ≥2)	1.367	0.523	6.824	1	0.009	3.924	1.407~10.942
Incorporating CPF-C (≤82.64 *vs*. >82.64)							
Size of gallstones (≤1.95 *vs*. >1.95 cm)	2.121	0.648	10.716	1	0.001	8.340	2.342~29.695
Course of gallstones (≤10 *vs*. >10 years)	2.299	1.002	5.263	1	0.022	9.960	1.398~70.974
CA199 (≤37 *vs*. >37 U/ml)	2.466	0.818	9.080	1	0.003	11.772	2.368~58.534
CPF-C (≤82.64 *vs*. >82.64)	1.243	0.504	6.082	1	0.014	3.467	1.291~9.312

Since CPF-B and CPF-C were collinear with age, age was excluded from the model. In addition to CPF-A, CPF-B, CPF-C, and age, factors included in the model include BMI, smoking, size of gallstones, course of gallstones, CEA, and CA199; all factors were included in the study as categorical variables (LR: forward).

### The evaluation of CPF-A, CPF-B, and CPF-C in the external validation samples

Then, we evaluated the diagnostic value of the three predictive models in the external validation samples. By comparing the AUC value of the ROC curves between the three indicators and their components, the results show that the AUC value of CPF-A, CPF-B, and CPF-C was still significantly higher than the five single factors of their components (*P* < 0.05). But there was no significant difference between the three predictive indicators (*P* > 0.05, **Table 7**). The C-index values of CPF-A, CPF-B, and CPF-C were 0.876, 0.873, and 0.854, and the corrected C-index values were 0.876, 0.873, and 0.853, respectively.

**Table 7:** Predictive values of predictive factors associated with GBC in the external validation samples.

Variable	AUC	Standard error	95% CI	*P*-value
CPF-A	0.876	0.030	0.817~0.935	<0.001
CPF-B	0.872	0.030	0.814~0.931	<0.001
CPF-C	0.854	0.035	0.785~0.924	<0.001
Age	0.748	0.042	0.666~0.831	<0.001
Size of gallstones	0.693	0.043	0.608~0.778	<0.001
Course of gallstones	0.722	0.047	0.631~0.814	<0.001
CEA	0.684	0.047	0.593~0.776	<0.001
CA199	0.704	0.048	0.611~0.798	<0.001

**Figure 6:**
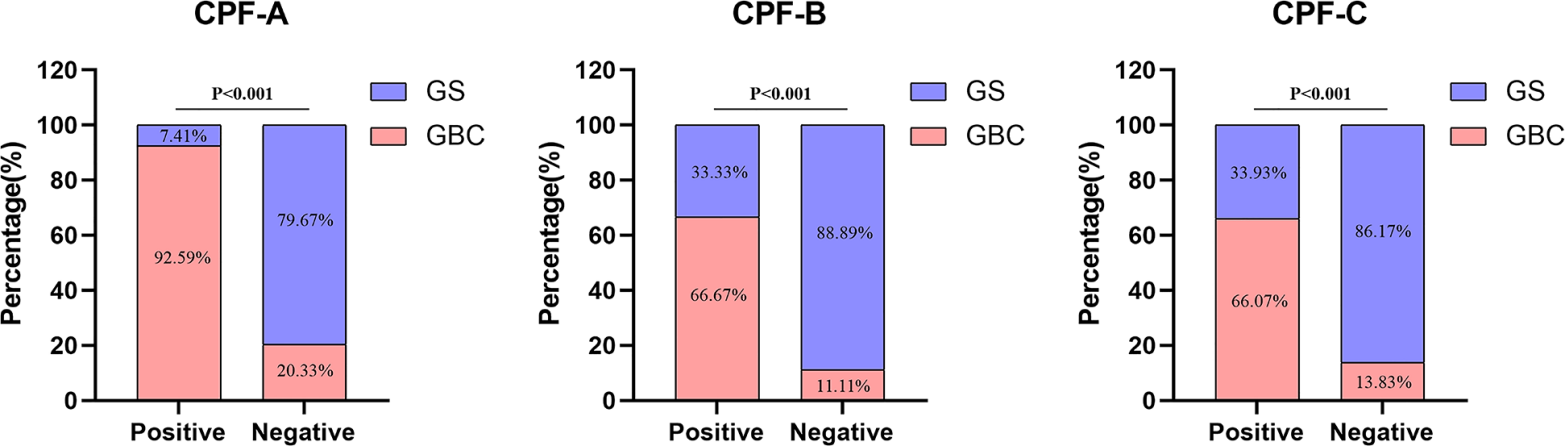
Diagnosis rate of CPF-A, CPF-B, and CPF-C in the external validation samples.

**Figure 7:**
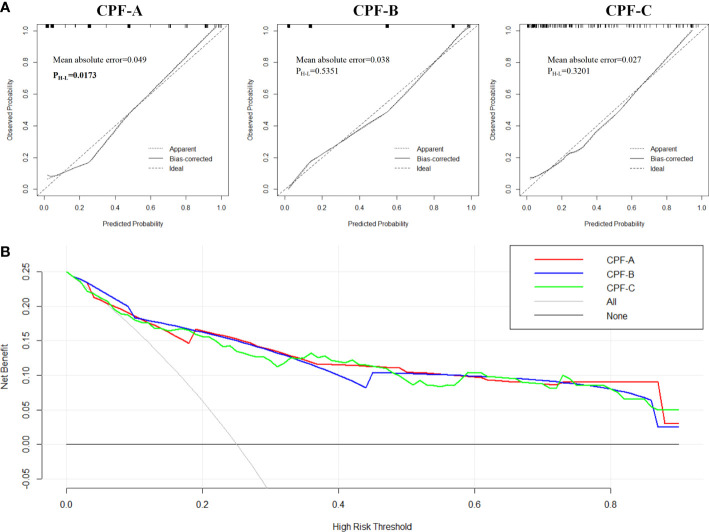
The evaluation of CPF-A, CPF-B, and CPF-C in the external validation samples. **(A)** Calibration curves of high-risk patients for GBC predicted by CPF-A, CPF-B, and CPF-C. **(B)** Decision curves of CPF-A, CPF-B, and CPF-C.

Next, we divided 150 patients that constituted the external validation samples into positive and negative groups according to the three predictive indicators. The results showed that there were 27 patients in the CPF-A-positive group, consisting of 25 GBC patients (92.59%) and 2 GS patients (7.41%), and 123 patients in the CPF-A-negative group, consisting of 25 GBC patients (20.33%) and 98 GS patients (79.67%). Therefore, in the external validation samples, the misdiagnosis rate of CPF-A was 7.41%, and the missed diagnosis rate was 20.33%. There were 60 patients in the CPF-B-positive group, consisting of 40 GBC patients (66.67%) and 20 GS patients (33.33%), and 90 patients in the CPF-B-negative group, consisting of 10 GBC patients (11.11%) and 80 GS patients (88.89%). Therefore, in the external validation samples, the misdiagnosis rate of CPF-B was 33.33%, and the missed diagnosis rate was 11.11%. There were 56 patients in the CPF-C-positive group, consisting of 37 GBC patients (66.07%) and 20 GS patients (33.93%), and 94 patients in the CPF-C-negative group, consisting of 13 GBC patients (13.83%) and 81 GS patients (86.17%). Therefore, in the external validation samples, the misdiagnosis rate of CPF-B was 33.93%, and the missed diagnosis rate was 13.83% (**Figure 6**). We then constructed the calibration curves (bootstrap = 10,000, *n* = 150), and the mean absolute errors of CPF-A, CPF-B, and CPF-C were 0.049, 0.038, and 0.027, respectively. Then, through the Hosmer–Lemeshow analysis, the *P*-values of CPF-B and CPF-C were 0.5351 and 0.3201, respectively (*P* > 0.05), which proved that the fitted value of CPF-B and CPF-C was in good agreement with the observed value. However, the *P*-value of CPF-A was 0.01725 (<0.05), which proved that there was a significant difference between the fitted value and the actual observed value (**Figure 7A**). Therefore, the fitting degree of CPF-A was poor.

Finally, we compared the diagnostic benefits of the three models by decision curve analysis. The results showed that there was no significant difference in the net benefit of CPF-A, CPF-B, and CPF-C in the external validation samples (**Figure 7B**). The results of the clinical impact curves showed that when the thresholds were approximately 0.4 or higher, the CPF-A, CPF-B, and CPF-C models predicted a high degree of agreement between the number of positives and the true number of positives (**Supplementary Material 2**).

## Discussion

GBC is a complex malignancy, and the mechanism of its occurrence is not very clear. An important role of laparoscopic cholecystectomy (LC) is to prevent the occurrence of GBC. However, due to the complexity of the causes of GBC, there is no consensus on the surgical indications for LC for preventive resection yet. Studies have reported that although LC has been promoted in many regions around the world, the incidence of GBC has only slightly decreased, suggesting that the current surgical indications for LC may also have certain defects (11). The occurrence of GBC is the result of multiple factors, and there are complex correlations between various factors. Therefore, the factors that really affect the occurrence of GBC are not yet clear. In addition, the gallbladder is located in a position difficult for imaging. The positive rate of traditional imaging methods for the diagnosis of GBC is not high. In this study, the diagnostic rate of ultrasound for GBC was only 62.2%, while contrast-enhanced CT and contrast-enhanced MRI examinations can increase the detection rate of GBC to 70%. Nevertheless, contrast-enhanced CT and contrast-enhanced magnetic resonance imaging as invasive examinations are difficult to implement as screening methods. Although many improved diagnostic strategies have been proposed in recent years, they are often expensive and cannot be implemented as screening methods. The abovementioned reasons provide great difficulty for the early diagnosis of GBC. Therefore, it may be particularly important to study the possible main factors affecting the occurrence of GBC and to establish screening strategies for the high-risk groups of GBC.

The increase in age is closely related to a high risk of GBC. According to previous reports, the median age of patients with GBC is 67 years, and the incidence rate is significantly higher when the age is over 75 years (1). Additionally, women have a high risk of GBC, and their incidence rate is 2–6 times higher than that of men. Estrogen and progesterone may be the cause of this difference (1). Estrogen may reduce the motility of the gallbladder and increase the risk of gallstone formation and biliary tract infection.

In the internal test samples of this study, the male:female ratio = 1:1.7 (88:150) in GBC patients, with a median age of 68 years, which is basically in line with existing reports, and shows that female gender and age >58.5 years were independent risk factors for predicting GBC (*P* < 0.05). However, we found that there was no significant difference in the sex ratio between patients with GBC and those without GBC among patients with gallstones (*P* > 0.05). Although it is true that the number of GBC in women is 1.7 times more than that in men, women are also 1.5 times more likely to have gallstones than men. This finding reveals that women have an overall higher risk of GBC, probably mainly due to the higher incidence of gallstones in women. In addition, although some studies (1, 3) have reported that smoking, occupational exposure, obesity, and diabetes may be related to the occurrence of GBC, the results of this study showed that none of them are independent risk factors affecting the occurrence of GBC (*P* > 0.05) and GBC cannot be well predicted by these factors.

Gallstones are the main risk factor for GBC. Approximately 80%-85% of GBC patients have gallstones (2). On the one hand, gallstones induce carcinogenesis by mechanically stimulating the gallbladder mucosa or mechanical obstruction to produce inflammation; on the other hand, the bacteria colonized by the biofilm on its surface degrade bile acids to produce toxic substances or induce bacteremia, inflammation, and chronic mucosal damage, which further causes cancer (2–4, 12, 13). However, the incidence of GBC in patients with gallstones is only 0.3%-3%, and in 66%-77% of the general population, gallstone disease is asymptomatic (3, 14). Therefore, gallstones prone to induce GBC may have certain characteristics. Because ultrasound is inaccurate in the detection of gallbladder thickness, and magnetic resonance, CT, and other tests are not routinely used as preoperative examination methods to detect gallbladder wall thickness, it is often difficult to accurately measure the preoperative gallbladder wall thickness in patients with gallstones. In addition, most GBC patients have uneven thickening of the gallbladder wall, and it is difficult to define the detection standard when it is used as a diagnostic basis. Therefore, we did not include gallbladder wall thickness as a factor in our study. Therefore, in this study, we explored the influence of factors such as the size of gallstones, the course of gallstones, and gallbladder atrophy on the occurrence of GBC (4).

The relationship between the size of gallstones and GBC has been confirmed by a series of studies. In clinical practice, it was generally believed that a single stone larger than 2-3 cm is more likely to induce GBC; thus, removal of the gallbladder is recommended (3, 15). This study showed that GBC may exist when the stone diameter is greater than 1.95 cm, and it is an independent risk factor for GBC in patients with gallstones (*P* < 0.05). The course of gallstones has also been reported to be associated with the occurrence of gallbladder cancer (16, 17). The results of this study showed that the incidence of gallbladder cancer was significantly increased in patients with gallstones with a course of more than 10 years, which was an independent risk factor for the occurrence of GBC (*P* < 0.05). In addition, although one study (14) reported that gallbladder atrophy is related to the occurrence of GBC, there is no significant correlation between gallbladder atrophy and GBC in patients with gallstones in this study. The reason may be that a considerable number of patients with gallstones also have gallbladder atrophy (7.5%), which is not very specific for the prediction of GBC.

Inflammation is closely related to the occurrence of GBC. Long-term chronic inflammation can cause DNA damage to the single columnar epithelial cells of the gallbladder mucosa, then cause repeated proliferation and repair of tissues, further inducing the release of cytokines as well as growth factors, and activate abnormal cell proliferation, eventually leading to GBC (18). In the study, compared with patients with gallstones, GBC patients showed higher levels of inflammation-related indicators, such as leukocytes, CRP, neutrophils, and lymphocytes, as well as multiple indicators related to liver injury and function, such as ALT, AST, and γ-GT. There were differences in albumin, ChE, ALP, etc. (*P* < 0.05). However, this phenomenon may be due to tumor-necrotizing inflammation or tumor invasion of the liver tissue surrounding the gallbladder bed. These indicators themselves have no clear diagnostic basis for the occurrence of GBC, so we did not include these indicators in the final multivariate analysis.

Effective tumor markers for the diagnosis of GBC are lacking. The classic indicators are CEA and CA199. This study showed that CEA and CA199 were still independent risk factors for predicting the occurrence of GBC (*P* < 0.05), and the cutoff value of the ROC curve was close to the classic cutoff value. However, the diagnostic sensitivity values of CEA (sensitivity 0.462, specificity 0.950) and CA199 (sensitivity 0.601, specificity 0.930) were not high, and they are still negatively expressed in a considerable number of GBC patients. Only relying on tumor indicators to screen GBC patients leads to misdiagnosis.

Because GBC is a disease caused by multiple factors, predicting the occurrence of GBC from a single angle is often not accurate. Therefore, we try to improve the stability of screening indicators for GBC by combining multifactor prediction. Based on the results of multivariate logistic regression analysis, we constructed three models and analyze their diagnostic ability. We included age (≤58.5 *vs*. >58.5 years), size of gallstones (≤1.95 *vs*. >1.95 cm), course of gallstones (≤10 *vs*. >10 years), CEA (≤5 *vs*. >5 ng/ml), and CA199 (≤37 *vs*. >37 U/ml) were combined to establish three new combined predictive factors (CPF). According to their construction methods, including the weighting method, integral method, and nomogram, we named them CPF-A, CPF-B, and CPF-C in turn.

Furthermore, we examined the diagnostic capabilities of the three indicators by different methods. Among the 438 patients (238 GBC+ 200 GS) that constituted the internal test samples, the number of positive patients for CPF-A, CPF-B, and CPF-C were 214, 282, and 245, respectively; the misdiagnosis rates were 12.15%, 20.92%, and 16.33%, and the missed diagnosis rates were 22.32%, 9.62%, and 17.1%, respectively. In the external validation samples consisting of 150 patients (50 GBC + 100 GS) from another medical center from a different region, the number of positive patients for CPF-A, CPF-B, and CPF-C were 27, 60, and 56, respectively. The misdiagnosis rates were 7.41%, 33.33%, and 33.93%, and the missed diagnosis rates were 20.33%, 11.11%, and 13.83%, respectively. Although the misdiagnosis rate of CPF-B and CPF-C is approximately 10%-20% higher than that of CPF-A, their missed diagnosis rate is approximately 10% lower than that of CPF-A. Moreover, in both the internal and external samples, the number of positive CPF-A indicators is less than the number of tumor patients in the samples, and the positive number in the external sample is only approximately 50% of the number of tumor patients, so the predicted value is difficult to cover the actual number of tumor patients. Therefore, as screening indicators, the actual value of CPF-B and CPF-C may be stronger than that of CPF-A.

Meanwhile, we observed the fit of the three indicators by constructing the calibration curves. When the *P*-value of the Hosmer–Lemeshow analysis is greater than 0.05, we consider the fit to be excellent. The results showed that although the fitting degree of the three indicators in the internal samples was good and their C-index values were close, in the external samples, the *P*-value of CPF-A is 0.01725 (<0.05), which proved that there is a significant difference between the observed value and the actual value, and the degree of fitting is poor. In addition, when the three predictors were included in the internal test samples again for multivariate analysis, CPF-A was not an independent risk factor for the occurrence of GBC in patients with gallstones (*P* > 0.05), while CPB-B and CPF-C were independent risk factors for GBC (*P* < 0.05). We came to the same conclusion after including these three indicators in the external validation samples. Therefore, CPF-B and CPF-C may be more powerful in predicting gallbladder carcinogenesis in patients with gallstones.

Furthermore, by establishing the DCA curves, we found that in the internal test samples, when the patient’s cancer risk fluctuates by approximately 30%-80%, the diagnostic power of CPF-C may be slightly better than that of CPF-B. However, this advantage is not shown in the small external validation samples, which may be the reason for the difference in the regression coefficients of the different samples. Moreover, the clinical impact curves showed that the accuracy of CPF-B and CPF-C diagnosis increased significantly when the patient’s cancer risk was higher than 40%-60%. Therefore, for high-risk cancer populations, both CPF-B and CPF-C have strong predictive power.

Finally, through the ROC curves, we found that the AUC values of CPF-B and CPF-C in both the internal test samples and the external validation samples were better than the rest of the individual indicators shown as independent risk factors (*P* < 0.05). At this time, the results obtained from the internal test samples showed that the cutoff value of CPF-B was 1.50, the AUC value was 0.899, the sensitivity was 0.937, and the specificity was 0.705. The cutoff value of CPF-C was 0.705, the AUC value was 0.912, the sensitivity was 0.861, and the specificity was 0.800.

These results suggested that when two or more of the five indicators, namely, age (≤58.5 *vs*. >58.5 years), size of gallstones (≤1.95 *vs*. >1.95 cm), course of gallstones (≤10 *vs*. >10 years), CEA (≤5 *vs*. >5 ng/ml), and CA199 (≤37 *vs*. >37 U/ml), were positive, or when the nomogram score was greater than 82.64, the risk of GBC in patients with gallstones was significantly increased. Therefore, for these patients, further in-depth examination or cholecystectomy may improve the early diagnosis rate of GBC and improve the survival of GBC patients.

There are still some shortcomings in this study. First, this study involved a total of 588 patients from two medical centers in different regions. The nomogram generated based on 438 test samples may have certain biases, so it failed to reflect more advantages in diagnostic capabilities than the point system. Further large-sample multicenter studies may reduce this bias and obtain a more accurate nomogram map. Second, this study was a retrospective study, and further prospective studies may be needed to explore the more practical application value of CPF-B and CPF-C.

## Conclusion

Age (≤58.5 *vs*. >58.5 years), size of gallstones (≤1.95 *vs*. >1.95 cm), course of gallstones (≤10 *vs*. >10 years), CEA (≤5 *vs*. >5 ng/ml), and CA199 (≤37 *vs*. >37 U/ml) were independent risk factors for GBC in patients with gallstones. When ≥2 indicators were abnormal (CPF-B), or the nomogram score based on risk factors was >82.64 (CPF-C), the risk of GBC was high. Both predictors have their own advantages. The simplicity and rapidity of CPF-B (point system) have irreplaceable advantages for the clinical judgment of high-risk groups. The CPF-C (nomogram) may bring more accurate prediction effects after the continuous evolution of modern technologies such as large network samples and artificial intelligence (AI). Surgical resection of high-risk patients by establishing early screening indicators for GBC may help reduce the mortality of this deadly disease and improve overall survival.

## Data availability statement

The raw data supporting the conclusions of this article will be made available by the authors, without undue reservation.

## Ethics statement

Ethical review and approval for the study of human participants was reviewed by the Zhangjiagang City First People’s Hospital Medical Ethics Committee. Written informed consent from the patients/next of kin was not required to participate in this study in accordance with the national legislation and the institutional requirements.

## Author contributions

The article topic design was contributed by KL and QL. ZZ contributed to the manuscript preparation and data analysis. BZ contributed to the plan of this subject. GW contributed to the manuscript editing. KG and PH contributed to the data collection. All authors contributed to the article and approved the submitted version.
