# Supplementary figures and images for: Risk factor analysis and construction of prediction models of gallbladder carcinoma in patients with gallstones
Source: Front Oncol. 2023 Feb 27;13:1037194. doi: 10.3389/fonc.2023.1037194 (PMC10009222; doi:10.3389/fonc.2023.1037194)

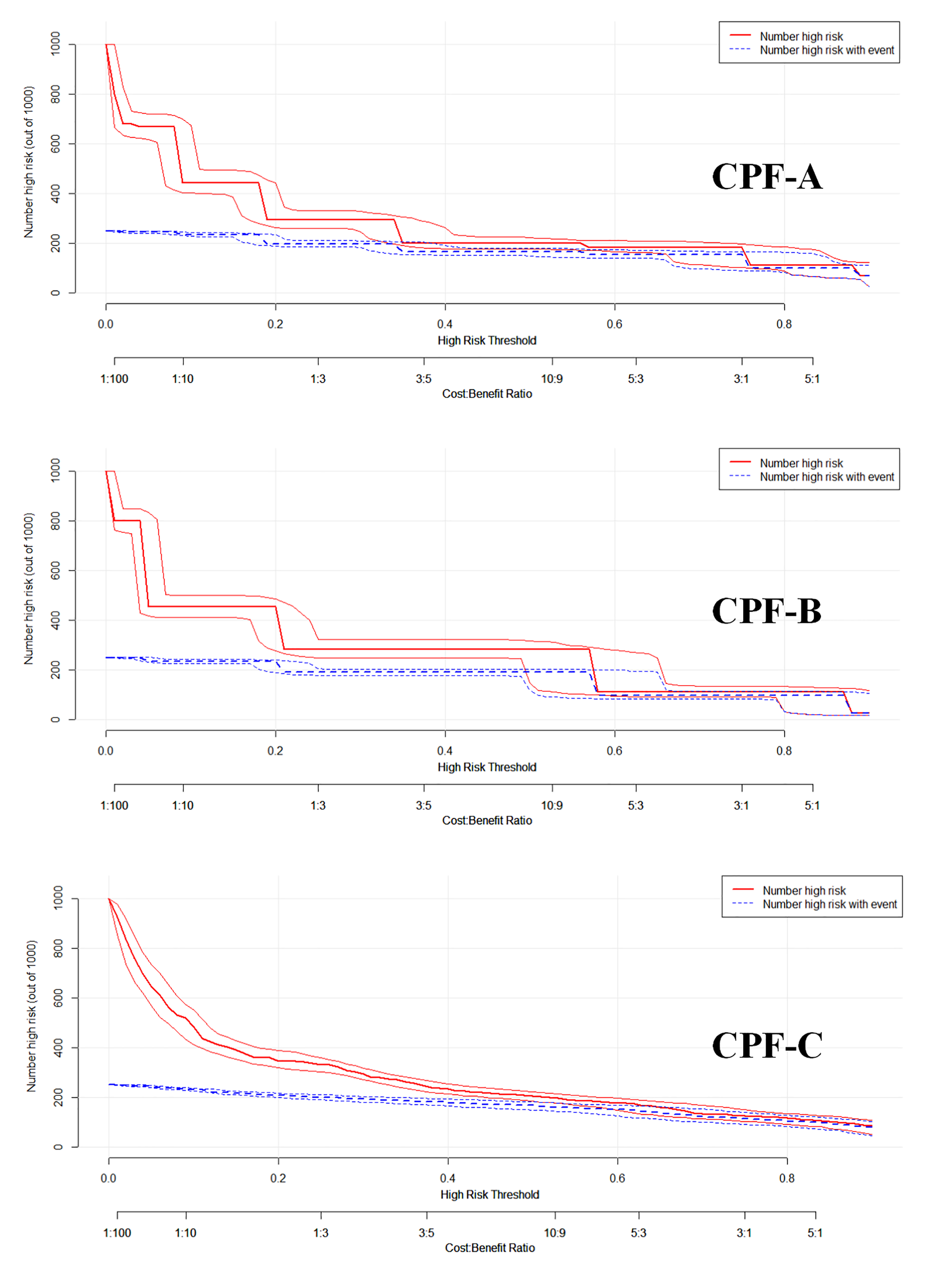

Supplement: Supplementary file 1 [file Image_1.tif]

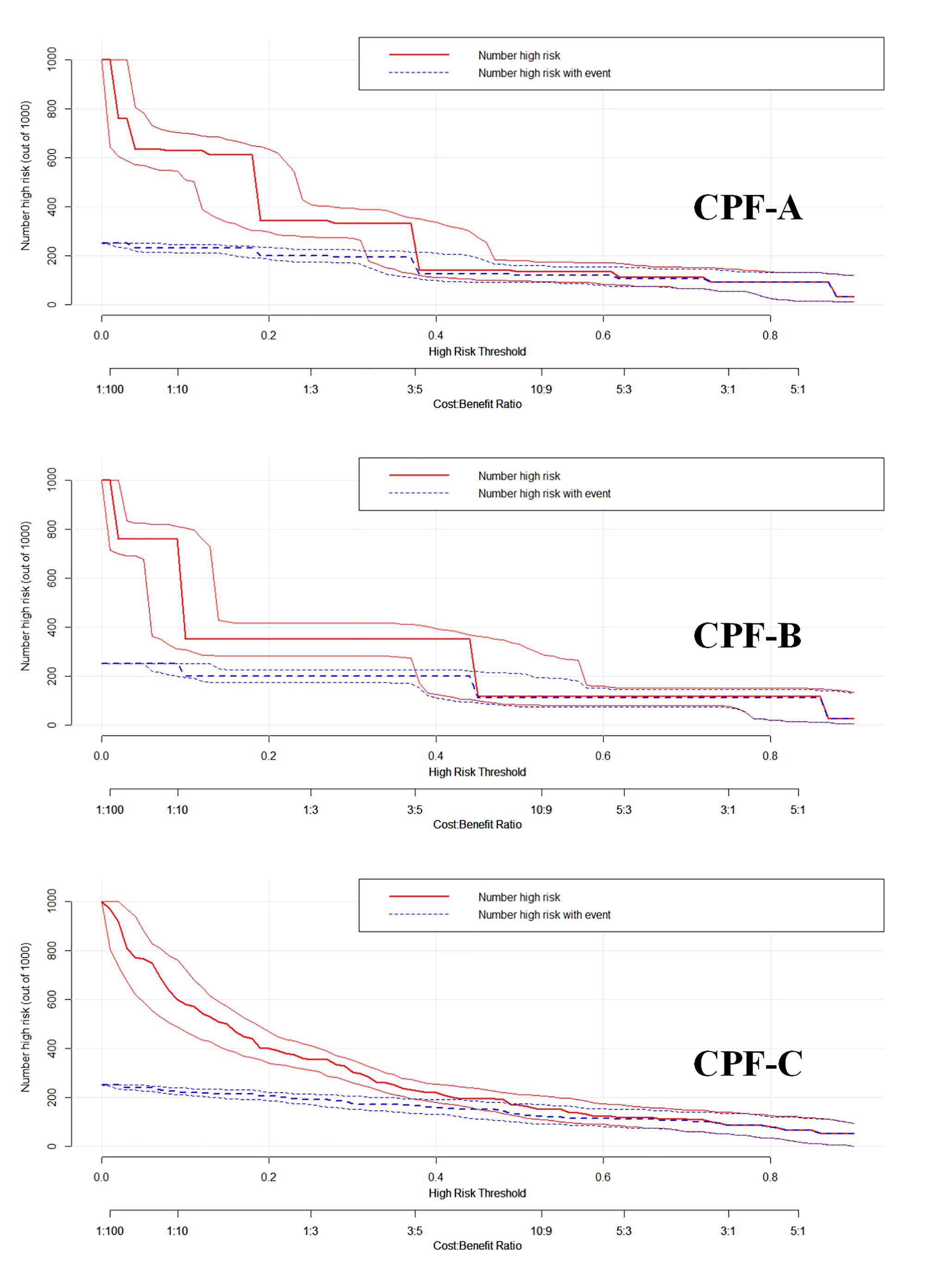

Supplement: Supplementary file 2 [file Image_2.tif]
